# Nitrogen-Doped Banana Peel–Derived Porous Carbon Foam as Binder-Free Electrode for Supercapacitors

**DOI:** 10.3390/nano6010018

**Published:** 2016-01-15

**Authors:** Bingzhi Liu, Lili Zhang, Peirong Qi, Mingyuan Zhu, Gang Wang, Yanqing Ma, Xuhong Guo, Hui Chen, Boya Zhang, Zhuangzhi Zhao, Bin Dai, Feng Yu

**Affiliations:** 1 Key Laboratory for Green Processing of Chemical Engineering of Xinjiang Bingtuan, School of Chemistry and Chemical Engineering, Shihezi University, Shihezi 832003, China; bzliushzu@sina.com (B.L.); qpeirong@sina.com (P.Q.); zhuminyuan@shzu.edu.cn (M.Z.); wanggang@shzu.edu.cn (G.W.); mayanqing@shzu.edu.cn (Y.M.); guoxuhong@ecust.edu.cn (X.G.); huichen_@outlook.com (H.C.); boai9594@outlook.com (B.Z.); ezuile@163.com (Z.Z.); daibin_bce@shzu.edu.cn (B.D.); 2 Institute of Chemical and Engineering Sciences, Agency for Science, Technology and Research, Jurong Island 627833, Singapore; zhang_lili@ices.a-star.edu.sg; 3 Engineering Research Center of Materials-Oriented Chemical Engineering of Xinjiang Production and Construction Corps, Shihezi 832003, China; 4 Key Laboratory of Materials-Oriented Chemical Engineering of Xinjiang Uygur Autonomous Region, Shihezi 832003, China

**Keywords:** banana peel, porous carbon foam, binder free, nitrogen doping, supercapacitor, energy conversion and storage

## Abstract

Nitrogen-doped banana peel–derived porous carbon foam (N-BPPCF) successfully prepared from banana peels is used as a binder-free electrode for supercapacitors. The N-BPPCF exhibits superior performance including high specific surface areas of 1357.6 m^2^/g, large pore volume of 0.77 cm^3^/g, suitable mesopore size distributions around 3.9 nm, and super hydrophilicity with nitrogen-containing functional groups. It can easily be brought into contact with an electrolyte to facilitate electron and ion diffusion. A comparative analysis on the electrochemical properties of BPPCF electrodes is also conducted under similar conditions. The N-BPPCF electrode offers high specific capacitance of 185.8 F/g at 5 mV/s and 210.6 F/g at 0.5 A/g in 6 M KOH aqueous electrolyte *versus* 125.5 F/g at 5 mV/s and 173.1 F/g at 0.5 A/g for the BPPCF electrode. The results indicate that the N-BPPCF is a binder-free electrode that can be used for high performance supercapacitors.

## 1. Introduction

Supercapacitors, also known as ultracapacitors or electrochemical capacitors (ECs), have attracted significant attention since the first patent filed in 1957 followed by successful commercialization for hybrid electric vehicles (HEVs) in the 1990s [1]. *Versus* conventional capacitors and Li-ion batteries, supercapacitors offer superior performance including high power capability, good operating voltage, long cycle life (>100,000 cycles), low cost, low maintenance, superior safety, environmentally benign, and fast charge propagation dynamics [2,3]. Recently, supercapacitors have shown advantages over other electrochemical energy storage (EES) devices in many fields requiring high reliability and short load cycle, including portable electronic devices, electric vehicles (EVs), memory back-up systems, *etc.*

Porous carbon along with metal oxides and conductive polymers is the most widely used electrode for supercapacitors because it offers a large surface area, low cost and easy processing [4,5,6]. Porous carbon offers a high capability for charge separation/accumulation at the electrode/electrolyte interface, depending on the charge-storage mechanism [7,8]. Generally, porous carbon is derived from organic molecules (e.g., acetonitrile) [9], polymers (e.g., polypyrrole) [10], meta-aminophenol formaldehyde resin [11], monolithic carbide [12], *etc.* This often involves synthetic steps using toxic reagents and complicated synthesis procedures [13]. These concerns as well as requirements for tailored materials have led scientists to develop sustainable, cheap, safe and environmentally friendly porous carbon for use as supercapacitor electrodes.

Here, we report nitrogen-doped banana peel–derived porous carbon foam (N-BPPCF) for use as a binder-free electrode for supercapacitors. The high porosity provided by the framework of the banana peel (BP) offers a high specific surface area and suitable pore size distribution for efficient contact between the electrolytes and the active materials. This, in turn, provides more active sites for electrochemical reactions and outstanding specific capacitance values [14,15,16]. To the best of our knowledge, this is the first report to describe the use of banana byproducts to generate carbon foam as an electrochemical reagent. This has significant implications for both the chemical and environmental community and is an excellent example of green synthesis.

## 2. Results and Discussion

**Figure 1:**
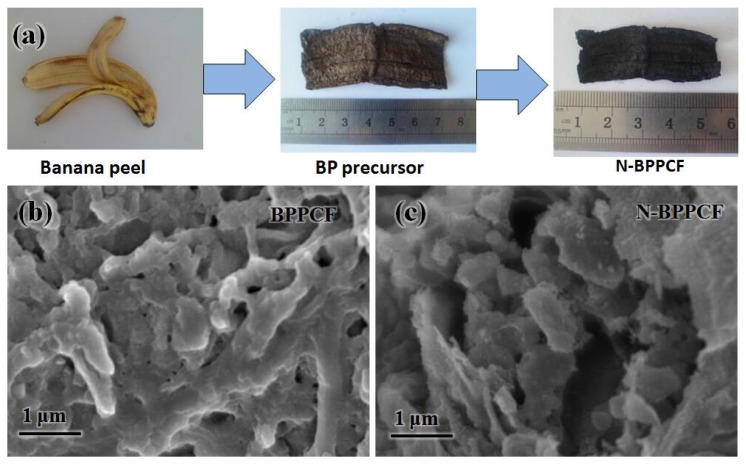
(**a**) Synthesis of porous carbon foam from banana peels. Scanning electron microscopy (SEM) images of (**b**) banana peel-derived porous carbon foam (BPPCF) and (**c**) nitrogen-doped BPPCF (N-BPPCF).

To understand the formation mechanism of N-BPPCF, a schematic illustration is proposed in Figure 1a. The pristine BP was firstly air-dried, hydrothermally heated and freeze-dried to yield a brown BP precursor. This precursor has a ribbon pattern-like structure 6 cm long and 2 cm wide, similar to non-processed BP. After the carbonization and nitrogen doping, black N-BPPCF was created with a length of 4.5 cm and a width of 1.5 cm.

**Figure 2:**
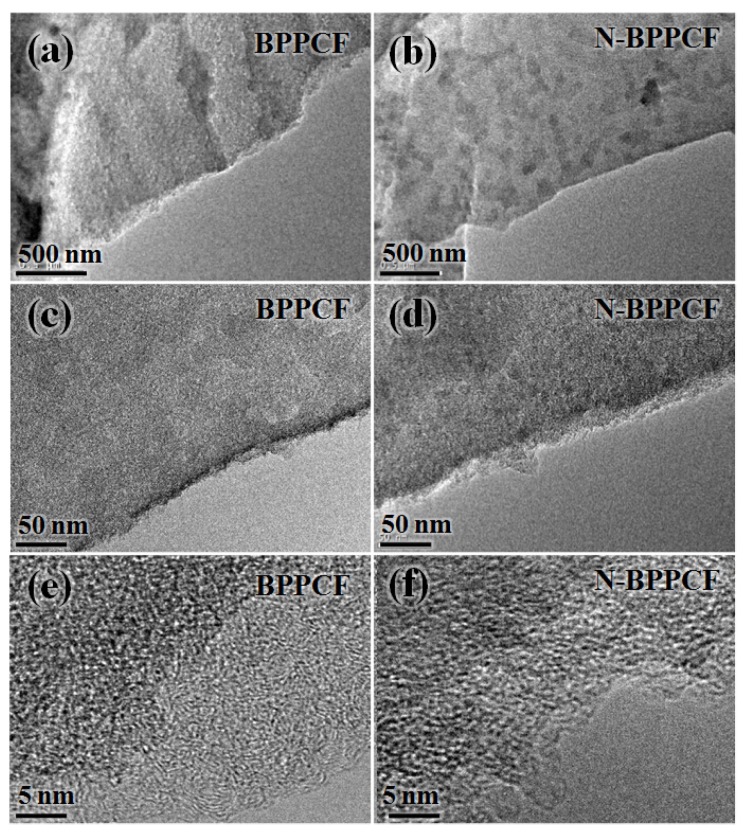
Transmission electron microscopy (TEM) and high-resolution TEM (HRTEM) images at different magnifications of (**a**, **c** and **e**) BPPCF and (**b**, **d** and **f**) N-BPPCF.

**Figure 3:**
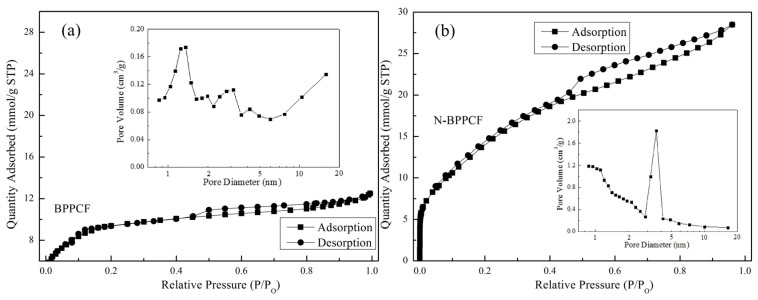
Nitrogen sorption isotherms of (**a**) BPPCF and (**b**) N-BPPCF, and the corresponding pore size distribution (insets).

Figure 1b,c shows typical scanning electron microscopy (SEM) images of the BPPCF and N-BPPCF porous carbon foam morphology, respectively. We used transmission electron microscopy (TEM) and high-resolution TEM (HRTEM) to further investigate the microstructure details of the BPPCF and N-BPPCF superstructures. Figure 2 shows TEM and HRTEM images at different magnifications of BPPCF and N-BPPCF. Both samples showed a porous structure with a possible pseudographite phase (Figure 2e,f). Moreover, the N-BPPCF produces a high specific surface area (SSA) of 1357.6 m^2^/g, a pore volume of 0.77 cm^3^/g and a Barrett-Joyner-Halenda (BJH) adsorption average mesopore size distribution around 3.9 nm (Figure 3 and Table 1). This is because the additional NH_3_ treatment at 900 °C further activates the carbon [17]. The N-BPPCF exhibited higher SSA and bigger pore volume than those of BPPCF. The porous-structured N-BPPCF offers good contact with electrolytes, and these pores strongly favor immediate electron and ion transmission [18].

**Table 1:** Specific surface area, porosity parameters and nitrogen content of the BPPCF and N-BPPCF samples. In the table, *S*_BET_ stands for BET surface area, while BJH pore diameter, total pore volume and meso-pore valume were abbreviated as *D*_BJH_, *V*_TPV_ and *V*_meso_.

Samples	*S*_BET_ (m^2^/g)	*D*_BJH_ (nm)	*V*_TPV_ (cm^3^/g)	*V*_meso_ (cm^3^/g)
BPPCF	648.3	4.22	0.4183	0.1903
N-BPPCF	1357.6	3.92	0.7651	0.1860

**Figure 4:**
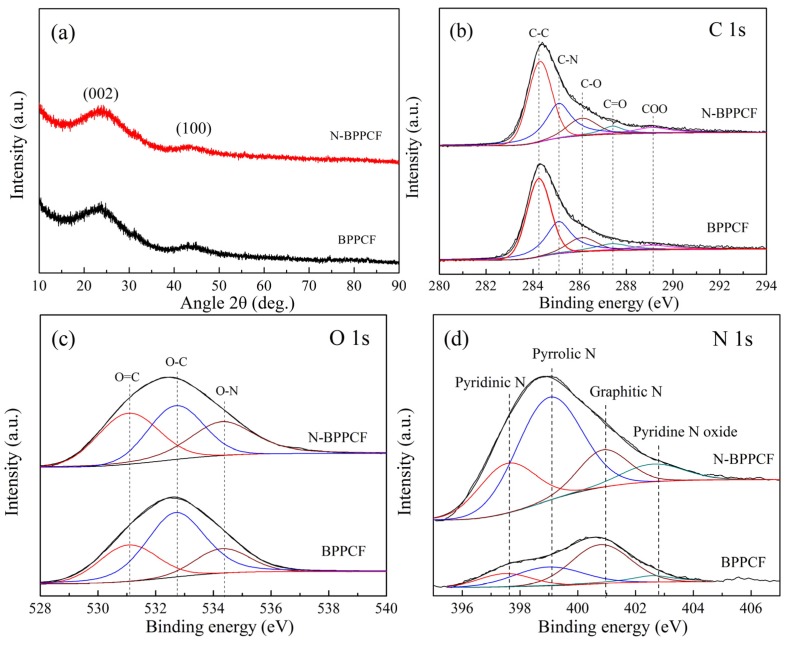
(**a**) X-ray diffraction (XRD) patterns for BPPCF and N-BPPCF samples. X-ray photoelectron spectroscopy (XPS) spectra of (**b**) C 1s, (**c**) O 1s and (**d**) N 1s for as-obtained BPPCF and N-BPPCF.

Figure 4a shows the XRD patterns of BPPCF and N-BPPCF. There is a broad peak at 2θ of about 23° corresponding to the (002) plane reflection of graphite. In addition, there is a small shoulder peak that appears at 2θ of 44° which corresponds to the (100) plane reflection of graphite. These two broadening peaks reveal the possible presence of the amorphous phase [19,20] and possible pseudographite nature [21] within the carbonaceous BPPCF and N-BPPCF.

XPS analysis of C, O, and N content in the as-obtained BPPCF and N-BPPCF shows three peaks around 284.2, 288.4 and 444.0 eV, as shown in Figure 4b–d. In the C 1s spectra of the samples, the sharp peak at 284.3 eV corresponds to sp2 carbon atoms. The peaks at 286.2, 287.5 and 289.2 eV are attributed to different C–O bonding configurations including C–O, C=O and C–OO bonds which decomposited from BP. Additionally, the peak at 285.1 eV from BPPCF indicates the presence of a C–N bonding configuration degraded mainly from organic compound amino acids in banana peels. Similarly, the bonding configurations of oxygen atoms in samples were characterized by high-resolution O 1s spectra. The O 1s signal consists of three distinct peaks—O=C, O–C and O–N, respectively. The corresponding binding energy of O 1s at 531.1 eV was assigned to O=C, at 532.7 eV to O–C and at 534.4 eV to O–N. All the different oxygen species were formed after thermal annealing of BP. The oxygen content in N-BPPCF decreases slightly with the increase of the nitrogen atomic percentage since the BPPCF was treated by ammonia gas. In the high resolution N 1s spectra, the peak can be attributed to the intensities of four components, such as pyridinic N (397.6 eV), pyrrolic N (399.1 eV), graphitic N (401.0) and pyridine N oxide (402.8 eV). The total nitrogen content in N-BPPCF was 8.7% higher than that of BPPCF (4.2%), as shown in Table 2. In N-BPPCF, pyridinic N constitutes 22.6 at. %, quaternary N constitutes 53.3 at. %, pyrrolic N consititues 15.3 at. % and pyridine N oxide constitutes 8.8 at. % (Table 3). Due to the doped nitrogen atoms acting as functional groups, the N-BPPCF has good hydrophilicity of the surface and easily contacts with electrolytes [17].

**Table 2:** Element composition by XPS of the BPPCF and N-BPPCF samples.

Samples	C (at. %)	O (at. %)	N (at. %)
BPPCF	90.27	5.52	4.21
N-BPPCF	86.29	5.04	8.67

**Table 3:** Atomic ratio of various N species from deconvolution N 1s spectra.

Samples	Pyridinic N	Pyrrolic N	Graphitic N	Pyridine N Oxide
BPPCF	14.51	27.35	49.13	9.01
N-BPPCF	22.61	53.27	15.28	8.84

**Figure 5:**
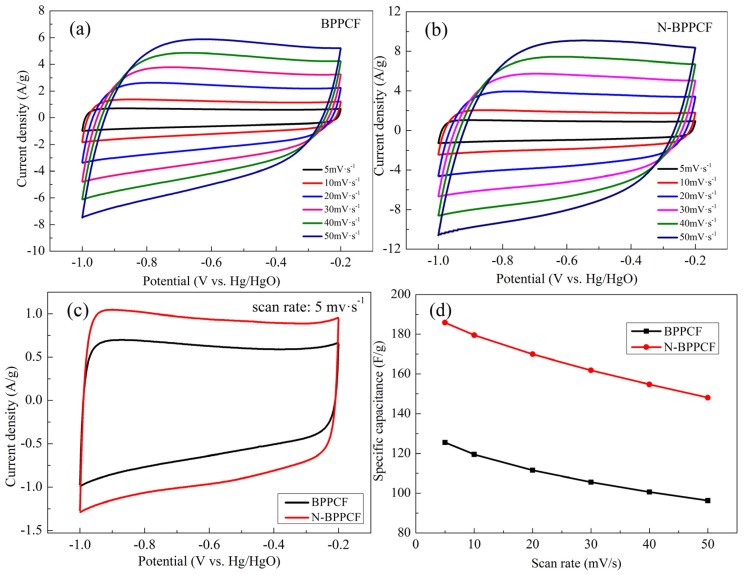
Cyclic voltammogram (CV) curves for (**a**) BPPCF and (**b**) N-BPPCF in 6 M KOH at different scan rates. (**c**) CV curves of the samples at a scan rate of 5 mV/s. (**d**) Specific capacitance calculated according to CVs.

Figure 5 presents cyclic voltammetry (CV) studies used to investigate the electrochemical properties of electrodes. Figure 4c shows CV performance of the BPPCF and N-BPPCF samples in 6 M KOH at the scan rate of 5 mV/s. The CV curves of both samples were rectangular, which is attributed to an ideal capacitance behavior of a porous carbon electrode. The rectangular shape of CV curves is not seriously distorted, even at high scan rates (Figure 5a,b). This indicates the porous carbon is suitable for aqueous electrolytes and that there is little concentration polarization within the pores due to ion transport limitations [22].

The N-BPPCF offers high specific capacitance (185.8 F/g at 5 mV/s) *versus* 125.5 F/g at 5 mV/s for BPPCF (Figure 5d). With increasing scan rates, the N-BPPCF shows smaller discharge capacitance, such as 179.5 F/g at 10 mV/s, 169.9 F/g at 20 mV/s and 161.9 F/g at 30 mV/s. Even at the scan rate of 40 mV/s and 50 mV/s, the N-BPPCF delivered discharge capacitance of 154.7 F/g and 148.0 F/g, respectively. This is 83.3% and 79.7% of the maximum capacitance at 5 mV/s. It was easy for N-BPPCF to achieve specific capacitance values over 140 F/g—the BPPCF was limited to below 130 F/g. This is principally because the N-BPPCF offers a specific surface area that, in turn, increases the capacitance [8,11,19].

**Figure 6:**
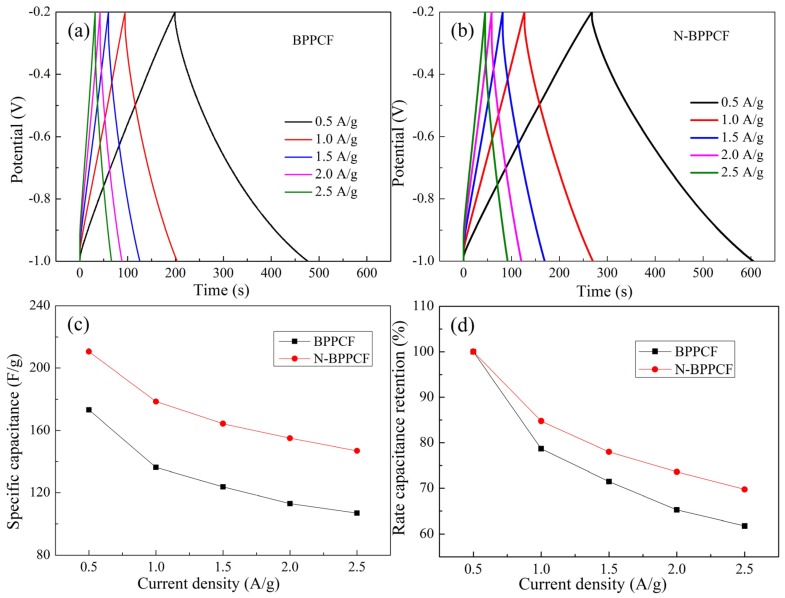
Galvanostatic charge/discharge (GCD) curves for (**a**) BPPCF and (**b**) N-BPPCF in 6 M KOH at various current densities. (**c**) Specific capacitance and (**d**) the corresponding rate capacitance retention of BPPCF and N-BPPCF.

We used galvanostatic charge/discharge (GCD) measurements to further investigate the electrochemical performance of the BPPCF and N-BPPCF at various current densities (Figure 6). The charge/discharge curves of both samples are linear and symmetrical without any infrared spectroscopy (IR) drop. However, the N-BBPCF offers a high specific capacitance of 210.6 F/g and 178.5 F/g, respectively, at 0.5 A/g and 1.0 A/g, respectively. These are much larger than 173.1 F/g and 136.3 F/g, respectively, for BPPCF. At the current density of 1.5 A/g and 2.0 A/g, the N-BPPCF can still deliver discharge capacitance of 164.3 F/g and 155.0 F/g. This confirms the excellent rate capabilities, with 78.0% and 73.6% of the maximum capacitance (210.6 F/g at 0.5 A/g). Even at the high current density of 2.5 A/g, the discharge capacitance of 146.9 F/g can be achieved. The corresponding obtained capacitance retention of N-BPPCF is 69.8% which is superior to 61.7% of BPPCF (Figure 6d). All data indicated that the N-BPPCF has great electrochemical performance that is superior to that of BPPCF. Of note, the results are comparable to those reported in literature, such as 175 F/g at 0.5 A/g in 6 M KOH [11] and 212 F/g at 0.5 A/g in 6 M KOH [13].

**Figure 7:**
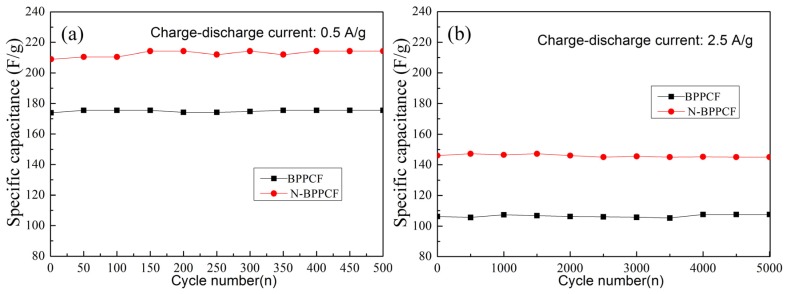
Cycle life of BPPCF and N-BPPCF at various currents: (**a**) 0.5 A/g for 500 cycles and (**b**) 2.5 A/g for 5000 cycles.

Figure 7 shows the capacitance of BPPCF and N-BPPCF. Both samples exhibited good reversible capacitance with a capacitance retention rate of about 100% upon cycling and after 500 cycles at 0.5 A/g. Even at 2.5 A/g, N-BPPCF as well as BPPCF performed good cyclic capacitance retention of 100% after 5000 cycles. This underlines the excellent charge–discharge stability of the N-BPPCF as well as BPPCF.

The N-BPPCF architecture offers excellent performance and practical sample preparation for supercapacitors. Of course, bananas are one of most popular fruits worldwide. There are more than 100 million tons produced every year. This results in significant organic waste from the peels. Using this byproduct as an electrode material is both environmentally sensitive and powerful from an electrochemical perspective. This coincides with other work focused on biomass [23] including from bagasse [24], rice husk [25], dead leaves [26], paulownia flower [27], tamarind fruit shell [28], *etc.*

Furthermore, the as-prepared N-BPPCF has nitrogen-containing functional groups to enhance the capacity, surface wettability and electronic conductivity of carbon materials due to nitrogen doping [29,30,31]. The N-BPPCF was easily achieved by treating the BPPCF with ammonia to incorporate nitrogen-containing functional groups [32,33]. The as-prepared, binder-free N-BPPCF could be directly used as an electrode without any conductive additives or binders. Recently, interest in binder-free electrodes has grown due to their efficiency and activity [34,35]. We believe that N-BPPCF is a powerful new binder-free electrode for supercapacitors. It has significant potential for use as is or in similar superstructures using other porous carbon foams.

## 3. Experimental Section

The raw banana peel (BP) was air-dried, collected and put in a glass dryer prior to use. The air-dried BP (1.5 g) was firstly added into 50 mL deionized water and then transferred into a 100 mL Teflon autoclave and hydrothermally treated at 120 °C for 5 h. Hydrothermal BP was achieved after filtering and washing with deionized water for three times. Subsequently, the as-prepared hydrothermal BP was freeze-dried at −50 °C for 12 h to obtain BP precursor. The carbonization and nitrogen doping process was carried out in two steps. The as-prepared BP precursor was firstly calcined at 900 °C for 5 h in Ar atmosphere to obtained BP-derived porous carbon foam (BPPCF). Secondly, the as-obtained BPPC was reduced in NH_3_ atmosphere at 900 °C for 1 h and then denoted as nitrogen-doped BPPCF (*i.e.*, N-BPPCF).

The BPPCF and N-BPPCF formation mechanism were evaluated with X-ray diffraction (XRD) analysis on a Bruker D8 Advance X-ray diffractometer (Karlsruhe, Germany) with Cu Kα radiation (λ = 1.5406 Å). Scanning electron microscopy (SEM) images were performed on a Hitachi SU8010 microscope (Tokyo, Japan). Transmission electron microscopy (TEM) images were obtained with a Tecnai G2 F30 field emission transmission electron microscope (Hillsboro, OR, USA). Micromeritics ASAP 2020 BEET apparatus (Norcross, GA, USA) was employed to determine Barrett-Joyner-Halenda (BJH) pore structure and Brunauer-Emmett-Teller (BET) specific surface area. The X-ray photoelectron spectroscopy (XPS) data were obtained with an AMICUS/ESCA 3400 electron spectrometer (Manchester, UK) from Kratos Analytical using Mg Kα (20 mA 12 KV)radiation. The binding energies were referenced to the C 1s line at 284.8 eV from adventitious carbon.

The electrochemical performance of the as-obtained BPPCF and N-BPPCF was evaluated using a standard three-electrode cell. The electrochemical performance such as cyclic voltammetry (CV) and galvanostatic charge/discharge (GCD) curves were performed using a CHI 660E electrochemical workstation at ambient condition. To fabricate the working electrode in three-electrode configuration, the as-obtained samples were cut into squares with edge length of 10.0 mm. A platinum sheet (10.0 mm × 10.0 mm) and a Hg/HgO electrode was the counter electrode and the reference electrode, respectively. The potentials were reported relative to the Hg/HgO reference electrode and the electrochemical measurements of the electrodes were recorded after stabilization. CV measurements were carried out at ambient temperature using 6 M KOH aqueous solution as electrolyte, the potential scan rates ranged from 0.5 to 50 mV/s within a potential range of −0.2 to 1.0 V *vs.* Hg/HgO.

The specific capacitances of the electrodes can be calculated by using Equation (1) with the measured CVs and Equation (2) from the galvanostatic discharge branches, respectively [3,36].

(1) $$C=\frac{\int^{\text{​}}IdV}{\upsilon \times \Delta V\times m}$$

Here, *C* is the gravimetric specific capacitance (F/g), *I* is the current (A), *ν* is the scan rate (mV/s), Δ*V* is the potential (V) and *m* is the total mass (g) of the samples.

(2) $$C=\frac{I\times t}{\Delta V\times m}$$

Here, *C* is the gravimetric specific capacitance (F/g), *I* is the discharge current (A), Δ*V* is the potential (V), *m* is the total mass (g) of the samples, and *t* is the discharge time (s).

## 4. Conclusions

In conclusion, nitrogen-doped banana peel–derived porous carbon foam (N-BPPCF) was successfully prepared and used as a binder-free electrode for supercapacitors. The N-BPPCF shows excellent electrochemical performances including a high specific capacitance of 185.8 F/g at 5 mV/s using CV measurement and 210.6 F/g at 0.5 A/g using galvanostatic charge/discharge measurement. We hope that N-BPPCF architecture will offer an additional way to prepare the binder-free electrodes and it shows potential for the synthesis of many other porous carbon foams.
